# P-2157. Genomic Characterization of Group A Streptococcus Isolates Circulating among Children in Greece during 2023

**DOI:** 10.1093/ofid/ofae631.2311

**Published:** 2025-01-29

**Authors:** Athanasios Michos, Elizabeth Barbara Tatsi, Maria-Myrto Dourdouna, Charilaos Dellis, Aspasia Rizou, Theano Georgakopoulou, Georgios Paradeisis, Angeliki Stathi, Anastassios Doudoulakakis, George Kalogeras, Elisavet Bozavoutoglou, Levantia Zachariadou, Vasiliki Syriopoulou

**Affiliations:** First Department of Pediatrics, Medical School, National and Kapodistrian University of Athens, ‘Aghia Sophia’ Children’s Hospital, Athens, Greece, Athens, Attiki, Greece; First Department of Pediatrics, Infectious Diseases and Chemotherapy Research Laboratory, Medical School, National and Kapodistrian University of Athens, “Aghia Sophia” Children’s Hospital, Athens, Greece, Athens, Attiki, Greece; First Department of Pediatrics, Infectious Diseases and Chemotherapy Research Laboratory, Medical School, National and Kapodistrian University of Athens, “Aghia Sophia” Children’s Hospital, Athens, Greece, Athens, Attiki, Greece; First Department of Pediatrics, Infectious Diseases and Chemotherapy Research Laboratory, Medical School, National and Kapodistrian University of Athens, “Aghia Sophia” Children’s Hospital, Athens, Greece, Athens, Attiki, Greece; First Department of Pediatrics, Infectious Diseases and Chemotherapy Research Laboratory, Medical School, National and Kapodistrian University of Athens, “Aghia Sophia” Children’s Hospital, Athens, Greece, Athens, Attiki, Greece; National Public Health Organization, Athens, Attiki, Greece; Microbiology Department, “Aghia Sophia” Children's Hospital, Athens, Greece., Athens, Attiki, Greece; Microbiology Department, “Aghia Sophia” Children's Hospital, Athens, Greece., Athens, Attiki, Greece; Microbiology Laboratory, “P. & A. Kyriakou” Children's Hospital, Athens, Greece., Athens, Attiki, Greece; Microbiology Laboratory, “P. & A. Kyriakou” Children's Hospital, Athens, Greece., Athens, Attiki, Greece; Microbiology Laboratory, “P. & A. Kyriakou” Children's Hospital, Athens, Greece., Athens, Attiki, Greece; Microbiology Department, “Aghia Sophia” Children's Hospital, Athens, Greece., Athens, Attiki, Greece; First Department of Pediatrics, Infectious Diseases and Chemotherapy Research Laboratory, Medical School, National and Kapodistrian University of Athens, “Aghia Sophia” Children’s Hospital, Athens, Greece, Athens, Attiki, Greece

## Abstract

**Background:**

An increase in invasive Group A Streptococcus (iGAS) and non-invasive GAS cases has been reported worldwide, especially among children, since 2022. The aim of this study was the genetic characterization of GAS isolates in the pediatric population of Greece.

**Methods:**

GAS isolates from children with iGAS reported to National Public Health Organization (NPHO) and a representative number of GAS from non-invasive infections were prospectively collected during 01-12/2023. Isolates were examined for antimicrobial susceptibility with Etest and the *emm* types were determined by Sanger Sequencing. Whole-genome Sequencing (WGS) was performed to selected GAS isolates to detect the toxin profile regarding 47 virulence factors, among them 11 superantigens.

**Results:**

GAS isolates from 511 children [266/467 (57%) males, median (IQR) age: 5.6 (3.8-8.0) years] were tested. iGAS represented 66/511 (12.9%) of isolates and among them 9/66 (13.6%) deaths were recorded. The most common GAS isolation sites were the following: throat (294/511, 57.5%), ear infusion (84/511, 16.4%) and skin lesions (60/511, 11.7%). All isolates were sensitive to penicillin. The resistance rates in erythromycin, clindamycin and tetracycline were 25/511 (4.9%), 18/511 (3.5%), and 61/511 (11.9%), respectively. We detected 29 different *emm* types, with the most prevalent being *emm12* (232/511, 45.4%) and *emm1* (135/511, 26.4%). Among the deceased children with iGAS, the *emm1* (7/9, 77.8%) and *emm12* (2/9, 22.2%) types were detected. Toxins profiles were examined with WGS in 6 non-invasive GAS isolates, 11 iGAS isolates and 7 iGAS isolates from deceased children. All isolates contained the *speB* gene (24/24, 100%). The *speA, speC, ssa* genes were detected: in 3/6 (50%), 3/6 (50%), 1/6 (16.6%) non-invasive GAS isolates, in 4/11 (36.4%), 4/11 (36.4%) and 0/11 (0%) iGAS isolates and in 5/7 (71.4%), 2/7 (28.6%) and 1/7 (14.3%) isolates from deceased children.

**Conclusion:**

During 2023, although several *emm* types caused infections in Greek children, the most prominent types were *emm12* and *emm1*. Continuous clinical and genomic surveillance of GAS is needed to comprehend the GAS disease trends and inform prevention strategies.

**Disclosures:**

All Authors: No reported disclosures

